# Task-oriented exercise effects on walking and corticospinal excitability in multiple sclerosis: protocol for a randomized controlled trial

**DOI:** 10.1186/s13102-023-00790-5

**Published:** 2023-12-21

**Authors:** Zahra Moslemi, Eduardo A. Toledo-Aldana, Bruce Baldwin, Sarah J. Donkers, Janice J. Eng, Prosanta Mondal, Julia O. Totosy de Zepetnek, Josef Buttigieg, Michael C. Levin, Cameron S. Mang

**Affiliations:** 1 Faculty of Kinesiology and Health Studies, University of Regina, 3737 Wascana Parkway, Regina, SK S4S 0A1 Canada; 2 School of Rehabilitation Sciences, College of Medicine, University of Saskatchewan, 104 Clinic Place, Saskatoon, SK S7N 2Z4 Canada; 3 Centre for Aging SMART at Vancouver Coastal Health, Department of Physical Therapy, University of British Columbia, 2177 Wesbrook Mall, Vancouver, BC V6T 1Z3 Canada; 4 Clinical Research Support Unit, University of Saskatchewan, 3200 Health Science E-wing, Saskatoon, SK S7N 5B5 Canada; 5 Department of Biology, University of Regina, 3737 Wascana Parkway, Regina, SK S4S 0A1 Canada; 6 Department of Neurology and Anatomy and Cell Biology, College of Medicine, University of Saskatchewan, 107 Wiggins Road, Saskatoon, SK S7N 5E5 Canada

**Keywords:** Multiple sclerosis, Neurological rehabilitation, Motor function, Transcranial magnetic stimulation, Biomarkers

## Abstract

**Background:**

Multiple sclerosis (MS) is a degenerative disease of the central nervous system (CNS) that disrupts walking function and results in other debilitating symptoms. This study compares the effects of ‘task-oriented exercise’ against ‘generalized resistance and aerobic exercise’ and a ‘stretching control’ on walking and CNS function in people with MS (PwMS). We hypothesize that task-oriented exercise will enhance walking speed and related neural changes to a greater extent than other exercise approaches.

**Methods:**

This study is a single-blinded, three-arm randomized controlled trial conducted in Saskatchewan, Canada. Eligible participants are those older than 18 years of age with a diagnosis of MS and an expanded Patient-Determined Disease Steps (PDDS) score between 3 (‘gait disability’) and 6 (‘bilateral support’). Exercise interventions are delivered for 12 weeks (3 × 60-min per week) in-person under the supervision of a qualified exercise professional. Interventions differ in exercise approach, such that task-oriented exercise involves weight-bearing, walking-specific activities, while generalized resistance and aerobic exercise uses seated machine-based resistance training of major upper and lower body muscle groups and recumbent cycling, and the stretching control exercise involves seated flexibility and relaxation activities. Participants are allocated to interventions using blocked randomization that stratifies by PDDS (mild: 3–4; moderate: 5–6). Assessments are conducted at baseline, post-intervention, and at a six-week retention time point. The primary and secondary outcome measures are the Timed 25-Foot Walk Test and corticospinal excitability for the tibialis anterior muscles determined using transcranial magnetic stimulation (TMS), respectively. Tertiary outcomes include assessments of balance, additional TMS measures, blood biomarkers of neural health and inflammation, and measures of cardiorespiratory and musculoskeletal fitness.

**Discussion:**

A paradigm shift in MS healthcare towards the use of “exercise as medicine” was recently proposed to improve outcomes and alleviate the economic burden of MS. Findings will support this shift by informing the development of specialized exercise programming that targets walking and changes in corticospinal excitability in PwMS.

**Trial registration:**

ClinicalTrials.gov, NCT05496881, Registered August 11, 2022. https://classic.clinicaltrials.gov/ct2/show/NCT05496881. Protocol amendment number: 01; Issue date: August 1, 2023; Primary reason for amendment: Expand eligibility to include people with all forms of MS rather than progressive forms of MS only.

**Supplementary Information:**

The online version contains supplementary material available at 10.1186/s13102-023-00790-5.

## Background

Multiple sclerosis (MS) is a chronic, progressive neurological condition currently without a cure that affects over 90,000 Canadians and more than 2.8 million persons worldwide [1–3]. The disease is characterized by destruction of the myelin sheath, a fatty layer around neurons critical for transmission of neural signals [4]. Typically, MS is diagnosed between the ages of 20 and 49 years [5]. In relapsing-remitting MS, the most common form, myelin is destroyed unpredictably in ‘relapse’ periods that are followed by periods of remission. However, approximately 65% of people diagnosed with relapsing-remitting MS transition to secondary progressive MS within 25 years and experience an ongoing, progressive destruction of myelin and further neurodegeneration [4]. Also, 10–20% of people with MS (PwMS) experience the primary progressive form, which is usually diagnosed after the age of 40 without any early relapses or remissions [4]. Crucially, as the myelin sheath deteriorates and further neurodegeneration occurs, motor, sensory, and cognitive functions are lost [6].

Walking is one of the most disrupted and valued motor functions across all types of MS, although impairments tend to be greater in progressive forms due to marked weakness of the lower extremities [7–9]. Disease-modifying drugs are the primary treatment approach for PwMS [10], but the impact of these drugs on walking function is limited. For example, only one pharmaceutical has been shown to improve walking speed among PwMS [11]. In this work, nearly one third of PwMS treated with dalfampridine improved their walking speed by approximately 25% (95% confidence interval, 21.0–28.4%) [11].

Exercise is established as safe, feasible, and beneficial for people with all types of MS, including those with high levels of impairment [12–16]. Established physical activity guidelines for PwMS indicate that resistance training of major muscle groups and 30 min of moderate-intensity aerobic activity at least two days per week can improve muscular strength and aerobic capacity [17–19]. Participation in physical activity, such as exercise training, has increasingly been recommended for PwMS to manage symptoms, promote wellness, boost participation in activities of daily living, and optimize quality of life [20]. Nevertheless, there remains limited understanding of what the best approach to exercise is for PwMS in terms of achieving functional outcomes. While meta-analyses have concluded that exercise among PwMS is associated with statistically significant improvements in walking speed, the magnitude of such improvements vary from large and clinically important to trivial across studies [21–24]. Interpretation of findings from exercise research in PwMS is limited by the use of generic or ill-defined interventions, few comparisons between exercise approaches, minimal consideration of impairment level, and limited use of functional outcomes [21, 22]. As a result, the “key ingredients” required for exercise to improve functional outcomes, such as walking speed, for PwMS remain largely undocumented.

Exercise training for people with sudden-onset neurological conditions (e.g., stroke) often employs a task-oriented approach, which refers to repetitive, goal-directed task practice that targets specific deficits in motor function [25–27]. Like other approaches to exercise, task-oriented exercise provides a stimulus to improve fitness, but its emphasis is on the re-learning of skills that were lost due to neurological damage and/or associated disuse. Although initial work suggests that task-oriented exercise may improve walking function in PwMS [14, 28, 29], it is not known if task-oriented exercise is superior to other exercise approaches. Evidence supporting the use of task-oriented exercise in PwMS was obtained from studies that unsystematically included task-oriented elements within other exercise approaches [21, 22], used a pre-post design with no control intervention [30, 31], compared the task-oriented approach to a non-exercise control intervention only [14, 32], or did not evaluate follow-up effects [30, 31]. Moreover, considering the degenerative nature of the disease, it is unclear if the emphasis on re-learning skills in task-oriented exercise is important for PwMS to optimize function or if more generalized approaches to exercise provide similar outcomes.

Central nervous system (CNS) changes following exercise have been reported in a small body of work in PwMS. Most of this work considers global changes in CNS health, reflected by changes in systemic blood markers of neuroinflammation and neuroprotection [33, 34]. For example, MS is associated with high systemic levels of the pro-inflammatory cytokine tumor necrosis factor-alpha (TNF-α) [35], which may be decreased by participation in regular exercise [36]. Neurotrophic factors, a family of biomolecules involved in neuroprotection, are also commonly examined (e.g., brain-derived neurotrophic factor, BDNF), and evidence suggests that BDNF may increase with exercise in PwMS [34]. Neurofilament light chain (NF-L) [37, 38], a cytoskeletal protein that reflects neuronal damage when detected in systemic blood, may better characterize the progression of the disease [39, 40], with evidence suggesting that it is decreased with regular aerobic exercise [41]. Nevertheless, conflicting findings and variable exercise interventions (i.e., type, intensity, duration) have limited the conclusions that can be drawn about exercise effects on blood markers of CNS health in PwMS [16, 33, 34]. Importantly, the neurophysiological effects of task-oriented exercise may extend beyond global improvements in CNS health to also include more specific changes in neural pathways involved in the ‘re-learning’ of motor skills. Such changes in the CNS can be measured with transcranial magnetic stimulation (TMS) [42]. While TMS-based measurements have been explored in PwMS for their utility as markers of disease burden [43], they can also be used to examine specific experience- or learning-dependent changes in the excitability of neural pathways that may be targeted by skill-based activities used in task-oriented exercise.

The overall goal of this work is to accelerate the development of improved exercise approaches for PwMS by characterizing the distinct functional effects and mechanistic underpinnings of task-oriented exercise for walking. The objective of the study is to compare the effect of task-oriented exercise against generalized resistance and aerobic exercise and a stretching control intervention on walking and CNS function in PwMS. Our primary hypothesis is that task-oriented exercise focused on walking will result in greater improvements in walking speed in PwMS than the alternate exercise interventions. We postulate that these task-oriented effects on walking speed will be accompanied by increases in corticospinal excitability for the ankle dorsiflexors that are not elicited by the other exercise approaches. Finally, we expect that exercise effects on musculoskeletal and aerobic fitness and global markers of CNS health will be similar across task-oriented and generalized resistance and aerobic exercise approaches, but greater than effects elicited by the stretching control intervention.

## Methods/design

### Study design and setting

This study is a three-arm randomized controlled trial (RCT) conducted in the Canadian province of Saskatchewan. The study was designed as a superiority trial through a collaborative effort of interdisciplinary researchers, clinicians, and PwMS. Study settings include a research laboratory at the University of Regina and community exercise centre. Any future amendments to the protocols described below will be first discussed and approved by the research team and then communicated to the Institutional Research Ethics Board, the clinical trial registry, study participants, and academic journals as appropriate.

### Study participants

Participants are recruited through advertisements and information circulated throughout the University of Regina, community organizations (e.g., MS Canada), healthcare facilities (e.g., local tertiary rehabilitation centre), and the Office of the Saskatchewan MS Clinical Research Chair. The Office of the Saskatchewan MS Clinical Research Chair (MCL) oversees a database of PwMS in the province who have consented to be contacted about participating in future MS-related research.

In the enrolment stage, a study team member screens participants for eligibility and provides them with a written informed consent form. Prospective participants have at least 24 h to review the informed consent form and discuss it with a study team member. The written informed consent form is signed by the participant and returned to the study team in advance or at the outset of the first study assessment session. Consent is re-established verbally at the outset of all subsequent assessment sessions. To be eligible for participation in this study, individuals must be: older than 18 years of age, diagnosed with MS by a neurologist, have an expanded Patient-Determined Disease Steps Score (PDDS) between 3 and 6 (i.e., experiences gait impairment but is ambulatory with or without aid), and considered not sufficiently active to achieve substantial health benefits (i.e., Godin-Shephard Leisure-Time Exercise Questionnaire score < 24) [44]. Individuals will be excluded if they: are unable to provide consent, have experienced a relapse in the past three months (self-reported, neurologist-confirmed), or are deemed to have a high-risk for exercise-related harm by a Canadian Society for Exercise Physiology accredited Clinical Exercise Physiologist (CSEP-CEP). Participants are informed that they can withdraw their participation and data from the trial at any time. Additional information collected from participants upon joining the study include age, sex, gender (self-reported), MS type, disease duration, medications, 29-Item Multiple Sclerosis Impact Scale score [45], clinical magnetic resonance imaging results (if available), other health conditions, and contraindications to TMS.

### Study arm allocation

All consenting participants who have completed a baseline assessment are stratified into either a mild (PDDS of 3–4) or moderate (PDDS of 5–6) impairment category. Participants from each strata are then randomly allocated to one of the three study arms: task-oriented exercise, generalized resistance and aerobic exercise, or stretching control. Allocation is completed by an administrative assistant not involved in data collection or analyses. Randomization lists were computer-generated in small blocks to help achieve balance across groups. The randomization list was created prior to participant recruitment by a research team member not involved in data collection or analyses.

### Blinding

Given the nature of the intervention, only the outcome assessors involved in the data collection and data analysts are blinded to the study groups and unblinding will not occur. Nevertheless, study participants are told that activities are individualized and not informed of the different study arms. Interventions are also scheduled to avoid contact between participants of different study arms and participants are asked to not describe their activities to those outside of their exercise group. Although it is not possible to blind program instructors to the intervention that they deliver, instructors are not made aware of the study aim and hypothesis.

### Interventions

The study includes three interventions: ‘task-oriented’ exercise (experimental), ‘generalized resistance and aerobic’ exercise (comparison), and ‘stretching’ exercise (control). All interventions involve 60-minute in-person sessions delivered three times per week for 12 weeks in groups of two to four participants. The volume and duration of exercise training aligns with other MS-focused literature reporting changes in motor function following various exercise interventions [21, 22, 46]. Likewise, the group training approach is supported by work suggesting that PwMS enjoy social support during exercise [47].

Interventions are completed under the supervision of a qualified exercise professional (i.e., kinesiologist and/or CSEP-CEP) with a 4-year undergraduate degree in kinesiology (or equivalent post-secondary degree) and a minimum of six months prior experience working with people with neurological conditions. The supervising exercise professionals are trained to deliver the interventions according to the study design and are provided with participants’ baseline assessment results prior to the first intervention session to support preparation. Training of the exercise professionals was completed with a standardized programme and delivered by the same instructor. As the exercise professionals must make practical decisions on how to adapt approaches to the individual needs of each participant, the first one to three sessions of each intervention are considered part of the intake process whereby the exercise professional(s) become familiar with the participants and identify suitable activities that meet the intervention criteria. A study team member attends an intervention session in the sixth week of delivery to monitor and evaluate aspects of intervention fidelity.

All interventions involve the whole body but differ in focus and content (Table 1). The task-oriented exercise intervention focuses on walking with elements of tailored functional strengthening, balance, agility, and repetitive, skill-based task (i.e., walking) practice similar to the ‘Fitness and Mobility Exercise’ (i.e., FAME) program, an established, evidence-based exercise program for people with stroke [48, 49]. A key element of this intervention is that it is entirely comprised of weight-bearing activities that train coordinated, functional lower-extremity movements rather than isolating muscle groups. As all activities are completed in standing, all upper-extremity movements will simultaneously train postural control. The duration and frequency of sessions ensures that high volumes of repetitions can be achieved, and rest provided, features of training that are considered crucial for induction of experience-dependent neuroplasticity [50, 51].

**Table 1:** Overview of interventions and example exercises

	Task-oriented	Resistance + Aerobic	Stretching control
**Format**	• Station-based circuit • Whole body movements • Standing • Free/cuff weights	• Machine circuit • Isolated movements • Seated, supported • Constant resistance	• Group instruction • Isolated movements • Seated, supported
**Exercises**	• Sit-to-stands • Step ups • Toe lift weight shifts • Wall push-up • Marching arm raises	• Knee flexion/extension • Full leg extension • Hip abduction/adduction • Chest press/seated row • Shoulder press/pulldown	• Quad/hamstring stretch • Triceps surae stretch • Hip flex/ext stretch • Pec/deltoid stretch • Shoulder rolls
	• Overground walking (30 min)	• Recumbent cycling (30 min)	• Relaxation activities (30 min)
**Prescription**	• Up to 5 min of continuous repetitions • Progressive loading and movement complexity • RPE 5–6/10	• 1–3 sets, 8–15 reps • Progressive loading • RPE 5–6/10	• 1–3 sets, 20 s stretches, 8–15 reps for range of motion • Movements unloaded • RPE 1–2/10

Notes: These are examples activities and prescriptions, rather than the full interventions. RPE: rating of perceived exertion

The generalized resistance and aerobic exercise intervention uses machine-based resistance training and recumbent cycling. In contrast to task-oriented exercise above, all activities are performed in seated, non-weight-bearing positions. The intervention is designed to target major muscle groups and meet general fitness-based recommendations for resistance and aerobic exercise for PwMS [15, 19, 52, 53]. The stretching exercise intervention involves stretching and relaxation activities in supported, non-weight-bearing positions with no external loading. This intervention serves as a control for confounding variables such as physical conditioning gained via transportation to intervention and study sessions, social interaction, secondary lifestyle changes (e.g., diet, sleep), and potential placebo effects from regular interaction with an exercise professional.

In the task-oriented exercise and generalized resistance and aerobic exercise interventions, heart rates are recorded with a chest-strap heart rate monitor and step count determined using a FitBit Inspire placed on the ankle [54, 55]. For all interventions, the activities led by the exercise professional and the participants rating of perceived effort (0–10 scale) are recorded to ensure alignment with the study design. The study team documents any circumstances leading to discontinuation or modification of interventions (e.g., injury, worsening disease symptoms); however, participants are retained in the trial whenever possible to prevent missing data. If a participant misses an intervention session, a study team member contacts the participant as a ‘check-in’ via phone, text, or email depending on participant preference.

### Outcome measures

All outcomes are assessed at baseline, after completion of the 12-week intervention, and at a six-week retention time point (Table 2). At each time point, outcome measures are collected across three separate assessment sessions within a seven-day period. A study team member schedules the sessions and contacts participants 24 h prior to the first of the three sessions as a reminder. The assessment sessions include: (1) All clinical assessments conducted in a 90-minute session, (2) TMS and fitness-based measures obtained in a two-hour session, and (3) Blood samples collected in a 30-minute session.

**Table 2:** Schedule of study enrolment, intervention and assessments

	Study period					
	Enrollment	Allocation	Baseline A_x_	Intervention	Post A_x_	Retention A_x_
Time point		Week 0	Week 0	Weeks 1–12	Week 13	Week 18
**Enrolment**:						
Eligibility screen	×					
Informed consent	×					
Study group allocation		×				
**Intervention**:						
Task-oriented exercise				×		
Generalized exercise				×		
Stretching control				×		
**Assessments**:						
Baseline variables (See Table 2)		×				
Primary outcome:						
T25-FWT			×		×	×
Secondary outcome:						
TA aMT			×		×	×
Tertiary outcomes:						
Additional clinical measures			×		×	×
Additional TMS measures			×		×	×
Blood marker measures			×		×	×
Fitness measures			×		×	×
Exit surveys					×	×

Notes: T25-FWT: Timed 25-Foot Walk Test; TA aMT: tibialis anterior muscle active motor threshold; TMS: transcranial magnetic stimulation; A_x_: assessment

#### Clinical measures

Walking speed will be measured as time to complete (0-180 s) the Timed 25-Foot Walk Test (T25-FWT). Change in T25-FWT time from the baseline to post-intervention time point is the primary outcome of the study. The T25-FWT provides an assessment of mobility and lower-extremity function through a measurement of fast walking speed [56, 57] and is the most commonly used measure of walking function in PwMS [57] with evidence of strong validity, reliability, responsiveness, and clinical benchmarks in MS [56, 58–60]. Further clinical assessments are conducted to ensure a comprehensive clinical evaluation, including the Mini Balance Evaluation Systems Test (comprehensive balance) [61], the Modified Ashworth Scale (spasticity) [62], the 9-hole Peg Test (dexterity) [63], the Symbol Digits Modalities Test (cognitive processing speed) [64], a weekly self-report of falls, and the expanded PDDS score. Clinical assessments described above are completed by a blinded licensed Physical Therapist.

#### TMS measures

TMS measures are obtained to characterize corticospinal excitability and cortical inhibition for the tibialis anterior (TA) and first dorsal interosseous (FDI) muscles of the self-reported stronger limb. Change in corticospinal excitability for TA, reflected by the active motor threshold (aMT) expressed as a percentage of maximum stimulator output (%MSO) across baseline to completion of interventions, is the secondary outcome. Additional TMS measures collected for TA and FDI muscles include: resting motor threshold (rMT), motor evoked potential amplitude and latency at 120% rMT, ascending slope of the MEP stimulus-response curve (FDI only), MEP amplitude and latency at 125% aMT for TA and 155% aMT for FDI, and cortical silent period (CSP) duration.

#### TMS and EMG procedures

TMS measures are collected by blinded research assistants trained by the Principal Investigator (CSM). During TMS assessments, all muscle responses are recorded using surface electromyography (EMG). The areas of electrode placement are rubbed with abrasive gel to remove dead skin and cleaned with 70% isopropyl alcohol. Bipolar gel Ag-AgCl electrodes (22 mm^2^) are placed over the target muscle belly with a grounding electrode on a nearby bony prominence (i.e., medial malleolus, styloid process). All EMG signals are pre-amplified (×1000) and band-pass filtered at 10 − 1,000 Hz using PowerLab amplification and EMG Systems (AD Instruments, Colorado, USA). Data for all evoked potentials are sampled at 2,000 Hz and recorded from 100 ms before to 400 ms after stimulus delivery.

TMS is applied using a Magstim 200^2^ stimulator (Magstim, Whitland, UK) connected to a 110-mm, concave, double-cone coil or a 70 mm figure-of-eight coil (D70 Alpha Coil) for study of the TA or FDI muscles, respectively. Coils are positioned with anterior-to-posterior current flow. Sites near the estimated motor representations are explored to determine the stimulation site at which the largest amplitude MEPs are elicited at the lowest stimulation intensity (i.e., the hotspot). With Brainsight neuronavigation software (Rogue Resolutions, Montreal, CA), the optimal stimulation site is recorded and used to maintain coil position and orientation for all TMS delivery. During TMS delivery, interstimulus intervals are maintained at approximately 4–6 s throughout all measurement protocols. rMT and aMT are determined by finding the lowest stimulation intensity (%MSO) that evokes MEPs of at least 50 µV and 200 µV, respectively, in five out of ten consecutive trials [65]. For TMS in the active muscle condition, participants maintain a low-level background muscle contraction (~ 10% of maximum voluntary muscle activity). The participant’s maximal voluntary muscle activity for the target muscle (TA or FDI) is first determined by recording EMG, rectified and low-pass filtered at 3 Hz during maximal voluntary isometric contraction. The peak EMG obtained from two efforts with two minutes rest between attempts is recorded as the participants maximal voluntary EMG. For subsequent TMS measures collected in the active muscle state, participants use visual and verbal feedback to maintain target muscle activity within a band on the computer monitor marking 8–12% maximal EMG. Short rest periods of 15–30 s are provided every 5–10 stimuli. For subsequent TMS measures, the number and intensity of stimuli delivered for study of the TA muscle are purposefully limited based on pilot participant feedback regarding tolerability and comfort of receiving TMS via the double-cone coil.

Corticospinal excitability for TA is further explored at rest by delivering ten single-pulse stimuli at an intensity of 120%rMT and calculating the average peak-to-peak MEP amplitude. For FDI, a stimulus-response curve is constructed by delivering ten single-pulse stimuli in a random order at stimulus intensities ranging from 90–150%rMT in 10% increments (70 stimuli total). The stimulus intensity by MEP amplitude relationship will be plotted, fit with a sigmoidal curve, and the slope of the ascending portion of the curve calculated [66]. Average latency of MEP responses elicited during these protocols will also be determined.

Cortical inhibition is evaluated by measurement of the CSP. While participants maintain 10% of their maximal muscle activity (see above) in the target muscle, ten TMS pulses are delivered at 125%aMT for TA and twenty pulses at 155%aMT for FDI. The duration of the transient reduction in muscle activity following the MEP in the target muscle will be quantified as the CSP [43]. Average peak-to-peak amplitude and latency of MEPs elicited in these protocols will also be calculated.

#### Fitness measures

Lower-extremity strength and endurance is determined by number of repetitions completed in the 30-second Sit-to-Stand Test in a standardized chair [67]. The sit-to-stand movement is performed without use of the arms for those who are able, while others use their arms to assist the movements across all assessments. Upper-extremity strength is measured by peak isometric grip force for each hand [68]. Participants complete two trials with each hand interspersed with two minutes of rest. A maximal exercise test is conducted on a recumbent cross trainer (NuStep T5XR, Plymouth, UK) to assess peak oxygen uptake (V̇O_2_). After determining resting heart rate and blood pressure, participants complete a two-minute warm-up at a self-selected step rate and power output and then begin the test with a workload of 15 W. The workload is increased every minute by 5 W for those with PDDS scores of 5–6 and by 10 W for those with PDDS scores of 3–4. During exercise testing, the following measurements are monitored: expired O_2_ and CO_2_ concentrations and air flow via a metabolic cart (TrueOne 2400; ParvoMedics, Sandy, UT), heart rate via a chest-strap heart rate monitor (Polar Electro; Oy, Kempele, Finland), and Borg’s 6–20 scale rating of perceived exertion (RPE). The test is stopped and peak V̇O_2_ recorded when at least one of the following criteria are met: a plateau in V̇O_2_ and heart rate with further increase in workload, a respiratory exchange ratio > 1.1, a RPE > 17, an inability to maintain the target workload, and volitional exhaustion. The recumbent stepper was chosen for the exercise test to mitigate any differences that might arise due to specificity of training. Fitness measures are collected by blinded research assistants trained by the Principal Investigator, with graded maximal exercise tests conducted under the supervision of a CSEP-CEP.

#### Blood marker measures

Systemic blood markers to be measured are serum TNF-α [35], BDNF [34], and NF-L [37, 38]. Blood samples are collected by a licensed phlebotomist at an off-campus location by venipuncture from the antecubital fossa to a vacutainer tube with no additive. The samples are allowed to clot and then centrifuged at 2,000 g for 30 min at 4 °C. Serum is aliquoted, transported to campus on ice, and stored at -80 °C within two hours of collection. Concentrations of all analytes will be measured using assays with appropriate sensitivity and reliability. Given low concentrations of NF-L, ultrasensitive assays will be considered for analysis in addition to more standard enzyme-linked immunosorbent assays. Assays will be run by a study team member in a preliminary analysis once a third of the projected sample has completed the study. Additional samples will be run in a single, batched analysis following completion of all data collection. Further blood markers linked to MS disease progression and exercise (e.g., interleukin-6) may be added to the analysis protocol if sample volume permits.

#### Study feasibility and experience

We record adherence to interventions and assessments, missing data, and adverse events. At the end of the intervention period and at study completion, participants complete exit surveys that query acceptability of interventions and assessments. The exit surveys also include questions related to any physical activity performed outside of intervention sessions and between intervention completion and retention testing. We also intend to develop a complementary qualitative study of participant and exercise professional experiences and perceptions of delivery of task-oriented exercise programming to PwMS with specific consideration of initial impairment level (i.e., PDDS of 3–4 and PDDS of 5–6).

#### Adverse events

Any adverse events will be self-reported by the participants and/or reported by exercise professionals and study personnel. Adverse events will be reported to the Institutional Research Ethics Board as required and assessed by the study team for seriousness, expectedness and causality following the guidelines of the National Health and Medical Research Council position statement for monitoring and reporting of safety for clinical trials [69].

#### Statistical analysis and sample size calculation

Baseline data collection will include both demographic and MS-related information (Table 3). The primary statistical analysis will compare T25-FWT performance at the post-intervention time point between the three study arms using ANCOVA (analysis of covariance) with a between-groups factor (task-oriented, generalized resistance and aerobic exercise, stretching control) and adjusting for baseline T25-FWT performance [28]. Planned pairwise comparisons will directly test differences between each study arm. Comparison of the secondary outcome and all other outcome measures at the post-intervention time point across study arms will follow the primary analysis methods (i.e., ANCOVA adjusting for relevant baseline measure). Exploratory analysis of change in outcome measures across all time points and between study arms will use a linear mixed effects model with Time point, Study arm, and Baseline T25-FWT performance as fixed factors. Participant will be a random factor. The models will account for dependencies/correlations resulting from repeated measurements. Final exploratory analyses will re-run all statistical tests with data disaggregated by sex [70–72]. For all statistical tests, the alpha will be 0.05. Point and 95% confidence interval estimates for study arm differences will be determined. Participants will be analyzed in the study arm to which they were randomized (i.e., intention-to-treat principle). Multiple imputation will be used to minimize biased estimates from missing data with the analysis based on a missing at random assumption [73]. We will conduct an interim analysis when 50% of the sample has completed study procedures. This interim analysis will allow dissemination (i.e., conference presentations) of preliminary findings. There is no stopping rule for the trial because no serious adverse events from the intervention are anticipated.

**Table 3:** Baseline data collection variables

Variables
Age (years)
Sex
Gender (optional)
Height
Weight
BMI
Type of MS
Year of MS onset (e.g., first symptom)
Year of MS diagnosis (by a neurologist)
Most recent relapse (month/year)
Patient Determined Disease Steps
Walking aid or assistive devices
More affected side (upper and lower body)
Medications (including disease-modifying therapy)
29-Item Multiple Sclerosis Impact Scale
Clinical MRI availability
Other health conditions
Employment/Work status
Typical Day
Fall history past 7 days
Fall history complete/triggers
Godin Physical Activity/Leisure Questionnaire
TMS contraindications

Notes: BMI: body mass index; MS: multiple sclerosis; MRI: magnetic resonance imaging; TMS: transcranial magnetic stimulation

The sample size was calculated based on the primary hypothesis and outcome. Most prior work studying the effects of exercise on walking speed has used interventions that cannot be distinctly classified as task- or non-task-oriented exercise, and has not compared the effects of different types of exercise [21, 22]. Thus, our sample size calculation is informed by a study in which combined gait and dual-task training resulted in a greater improvement in walking speed than lower-extremity resistance exercise in PwMS [28]. The combined gait and dual-task training (n = 26) yielded an average improvement in walking speed of 21.4%, while the resistance exercise (n = 12) yielded only a 2.5% change (i.e., null) [28]. The effect size describing the difference between groups in change in walking speed was large and significant (Cohen’s d = 0.95, 95% CI: 0.2–1.7) [28]. As the proposed intervention is longer and of higher volume than the prior work [28], we expect an effect size of similar or greater magnitude. Thus, based on the effect size reported in the prior work [28], we determined that a total of 63 participants (n = 21 per study arm) will be required to detect a clinically important mean difference of 20% in walking speed on the T25-FWT [59, 74] between study arms at the post-intervention time point with a two-sided significance level of 5%, a power of 85%, and equal allocation to the three arms of the trial.

#### Monitoring and data management

This study, including the participant consent form, has received ethical approval from the University of Regina Research Ethics Board (REB file 2021 − 197). Given that this is a low-risk intervention, no data monitoring review committee is required; however, the University of Regina Research Ethics Board has the authority to audit the study at any time to ensure compliance with approved protocols. Meetings of the research team will be held every three to six months to discuss day-to-day management and organisation of the study, including participant recruitment, delivery of the intervention, and participant monitoring.

All data, including the final trial dataset, is de-identified, coded and stored on a University of Regina server that is accessed only by members of the study team from password-protected computers. Physical copies of the data recording sheets are stored in locked filing cabinets at the University of Regina. All data is checked regularly by the study team to ensure protocols and ethical guidelines for data collection and analysis are followed. Study-related documents will be archived at the University of Regina at the end of the study and stored for a minimum of five years according to current ethical guidelines.

#### Dissemination plan

Findings describing the primary outcome analysis will be reported in scientific publications, which will include results regardless of the direction or magnitude of the effect. The results will also be presented at national and international conferences that target researchers and healthcare providers. Authorship on scientific publications and conference presentations will be determined as per recommendations from the International Committee of Medical Journal Editors [75]. Dissemination of non-academic outputs (e.g., lay summaries and public presentations) will capitalize on partnerships with local exercise and rehabilitation centres and the Saskatchewan Division of the Multiple Sclerosis Society of Canada. Pending results, future creation of MS-specific task-oriented exercise resources could support program implementation. Access to de-identified data will be granted upon reasonable request.

## Discussion

There are approximately 90,000 PwMS in Canada [3] with the highest prevalence in the Prairie Provinces including Saskatchewan [76]. Beyond the direct effects experienced by PwMS, the economic burden on society via lost years of working life and healthcare costs is substantial [77]. The idea of a paradigm shift in MS healthcare towards the use of “exercise as medicine” has been proposed as a means to improve outcomes and alleviate the economic burden of MS [16]. Yet, ongoing work suggests that PwMS have difficulties engaging in physical activity and exercise [78, 79] and that there are limited exercise services that are specialized to their needs and goals [80]. Moreover, a “best” exercise approach for PwMS to optimize walking function and counter pathophysiological changes in the CNS is not well characterized. Research that compares exercise approaches and considers underlying CNS mechanisms in PwMS will advance the field towards providing targeted exercise prescriptions that maximize functional gains and related neural changes.

The positive effects of exercise on symptom management and overall health and wellness provide sufficient rationale for it to be recommended and prescribed to PwMS [20]. However, if a person with MS has a certain number of hours per week to dedicate to an exercise routine, currently it is not clear on what type of exercises that time is best spent. It is likely that the full power of exercise to benefit PwMS will not be realized without efforts to optimize an exercise approach to both the specific goals of PwMS and to the pathophysiology of the disease. Task-oriented exercise is an evidence-based approach to support functional gains and underlying neuroplasticity in people with stroke [14]. Although it is not a typical approach to exercise prescription for PwMS and other neurodegenerative conditions, initial findings suggest that it has potential for improving valued motor functions, such as walking [14]. Nevertheless, other work using a more generalized exercise approach has also demonstrated improvements in walking function compared to a non-exercise control intervention [12]. Here, we designed a novel and comprehensive study to determine the distinct effects of a task-oriented exercise approach relative to a more generalized exercise approach for improving walking function and eliciting neural changes in PwMS.

Several challenges and limitations need to be considered. The nature of the study prohibits indisputable blinding of participants and intervention instructors. Strategic scheduling of interventions and providing instructors only with necessary information mitigate potential confounds. The activities of participants outside of intervention scheduling and between intervention end and the retention time point also cannot be controlled, but information on extracurricular physical activity is collected in the study exit survey for consideration in results interpretation. Another consideration is that we plan to recruit participants with all types of MS, which present with different disease courses and potentially different neurobiology [4]; however, this approach is consistent with other related research [21, 22] and may improve generalizability of results. Given prior research suggesting limited retention of effects of exercise interventions on motor function [31], the long-term benefits of this work for PwMS may be questioned. It is plausible that the targeted nature of the task-oriented training employed in the current research will result in more persistent benefits than prior work. Nevertheless, it is likely that PwMS require more than a single 12-week intervention to maximize and retain functional improvements in the long-term. Instead, this work should be considered a critical step towards development of optimal intervention strategies that will require ongoing or cyclic delivery for the maximum benefit of PwMS. Finally, the limitations of our physiological outcome measures must also be acknowledged. TMS is an inherently variable technique that probes specific neural mechanisms [42] which overlap only partially with mechanisms of neuroplasticity that may support functional gains induced by task-oriented exercise [81]. Likewise, systemic blood markers may not precisely reflect changes occurring in the CNS [38].

Recent work highlighted the lack of evidence for exercise effects on functional outcomes and CNS change in PwMS [46]. The current study is guided by the mechanistic hypothesis that task-oriented exercise, relative to other exercise approaches, may maximize functional gains in PwMS by preferentially engaging experience- or learning-dependent changes in corticospinal excitability. Findings have potential to improve understanding of the best approach to improve walking function for PwMS.

### Trial status

Participant enrolment began in May 2022 and recruitment is ~ 30% complete. Note that the clinical trials registration with our planned recruitment start date was submitted prior to recruiting any participants. However, one participant was recruited but no participants started the intervention before the trial was officially registered (August 11, 2022). The trial is expected to be complete by January 2025.

### Electronic supplementary material

Below is the link to the electronic supplementary material.

Supplementary Material 1

Supplementary Material 2

## Data Availability

Access to de-identified data and other study materials will be granted upon reasonable request.

## Declarations

### Appendices

Informed consent materials.

## Abbreviations

aMT: active motor threshold
ANCOVA: analysis of covariance
BDNF: brain-derived neurotrophic factor
CSEP-CEP: Canadian Society for Exercise Physiology accredited Clinical Exercise Physiologist
CNS: central nervous system
CSP: cortical silent period
EMG: electromyography
FDI: first dorsal interosseous
MEP: motor evoked potential
MS: multiple sclerosis
%MSO: % maximum stimulator output
NF-L: neurofilament light chain
PDDS: Patient-Determined Disease Steps
PwMS: people with MS
RCT: randomized control trial
RPE: rating of perceived exertion
rMT: resting motor threshold
TA: tibialis anterior
TMS: transcranial magnetic stimulation
TNF-α: tumor necrosis factor-alpha
T25-FWT: Timed 25-Foot Walk Test
V̇O_2_: oxygen uptake
