# Supplementary material for: Task-oriented exercise effects on walking and corticospinal excitability in multiple sclerosis: protocol for a randomized controlled trial
Source: BMC Sports Sci Med Rehabil. 2023 Dec 21;15:175. doi: 10.1186/s13102-023-00790-5 (PMC10734154; doi:10.1186/s13102-023-00790-5)
Supplement: Supplementary file 2 — Supplementary Material 2 [file 13102_2023_790_MOESM2_ESM.pdf]

UNIVERSITY OF REGINA

Research Participant Information and Consent Form

Title of the study: Evaluating exercise effects on central nervous system function in people with multiple sclerosis

Researchers: Cameron Mang, PhD (Principal Investigator), Faculty of Kinesiology and Health Studies, University of Regina, (t) 306-585-4066, (e) cameron.mang@uregina.ca; Sarah Donkers, PT, PhD (Co-Investigator), School of Rehabilitation Sciences, University of Saskatchewan, (e) sarah.donkers@uregina.ca; Josef Buttigieg, PhD (Co-Investigator), Department of Biology, University of Regina, (e) josef.buttigieg@uregina.ca; Prosanta Mondal, PhD (Co-Investigator), Clinical Research Support Unit, University of Saskatchewan, (e) prosanta.mondal@usask.ca; Janice Eng, PhD (Co-Investigator), Department of Physical Therapy, University of British Columbia, (e) janice.eng@ubc.ca; Julia Totosy de Zepetnek, PhD (Co-Investigator), Faculty of Kinesiology and Health Studies, University of Regina, (t) 306-585-4370, (e) julia.totosy@uregina.ca; Michael Levin, MD, FAAN, FANA, College of Medicine, University of Saskatchewan, (e) michael.levin@usask.ca; Zia Rehman, MD, FRCPC(C), Dip ABPN (Neurology) (Collaborator), Regina General Hospital, (e) drzrehman@sasktel.net; Zahra Moslemi, BA, MSc, Graduate Student Research Assistant, University of Regina, (e) zmc816@uregina.ca; Heather Foy, Undergraduate Student Research Assistant, University of Regina, (e) hcf754@uregina.ca; Nathan Bye, Undergraduate Student Research Assistant, University of Regina, (e) ndb865@uregina.ca; Zahra Moslemi, Graduate Student Research Assistant, University of Regina, (e) zmc816@uregina.ca; Amir Yahya Raja, Graduate Student Research Assistant, University of Regina, (e) arl550@uregina.ca; Eduardo Toledo-Aldana, BSc (Kin), Graduate Student Research Assistant, University of Regina, (e) toledoae@uregina.ca; Kristen Plandowski, BSc (Kin) Graduate Student Research Assistant, University of Regina, (e) kep933@uregina.ca; Robert Kraakman, BSc (Kin), Graduate Student Research Assistant, University of Regina, (e) kraakmar@uregina.ca; Sherrise Richards, Administrative Assistant, University of Regina, (e) sherrise.richards@uregina.ca

---

Introduction:

You are being invited to participate in this research study because we are interested in determining how exercise can be best prescribed to benefit the central nervous system for people with multiple sclerosis (MS).

Before you decide to participate, it is important that you understand what the research involves. This consent form will tell you about the study, why the research is being performed, what will happen to you during the study, and the possible benefits, risks, and discomforts.

If you wish to participate, you will be asked to sign this form. Your participation is completely voluntary, so it is up to you to decide whether or not to participate in this study. If you decide to take part in this study, you are free to withdraw at any time without giving any reasons for your decision and your choice not to participate will not affect your relationship with any of the researchers or institutions conducting the research. Please take time to read the following information carefully and feel free to discuss it with your family, friends, and doctor or health professional before you decide.

Why is this study being done?

The purpose of the study is to improve understanding of how to best prescribe exercise to benefit central nervous system function for people with MS. Although exercise is known to be beneficial for managing many symptoms related to MS, its effects on the central nervous system, and whether these effects change with different types of exercise, are not well understood. This research will be the first to compare the effects of different types of exercise on central nervous system changes in people with MS.

Who can participate in this study?

You can participate if you: have received a diagnosis of primary progressive, secondary progressive, or relapsing-remitting MS by a neurologist, have been cleared to exercise by a physician, experience limitations in your mobility and activities of daily living due to MS, and are able to walk 25 feet with or without assistance. You cannot participate if you: have experienced a relapse of your MS within the last three months, have any history of neurological conditions other than MS (e.g., epilepsy, acquired brain injury), have received a psychiatric diagnosis, engage in substance abuse, or are highly physically active.

What does the study involve?

If you agree to participate in this study, you will provide information to the research team regarding your age, sex, gender, MS type, disease duration, other medical conditions, and medications. You will take part in a set of two assessments and provide a blood sample at the outset of the study, following a 12-week exercise intervention, and six weeks after the end of the exercise intervention. At the end of the exercise intervention and at the six week follow you will complete exit surveys that inquire about your experiences with the study. Each of the assessment sessions will require approximately 2 hours of your time. The assessments will include tests of your fitness, movement abilities including walking function, and central nervous system excitability using a technique called transcranial magnetic stimulation (TMS). TMS is a form of non-invasive brain stimulation. When the brain stimulation is delivered it will make a “click” noise and you will feel a tap on your head. It will also result in a twitch of a muscle in your hand or leg. The size of the twitches provides information about the excitability of the nervous system pathways that are activated by the stimuli.

During the 12-week exercise intervention, you will participate in 60-minute exercise sessions three times per week. The sessions will be delivered either at the University of Regina Centre for Health, Wellness and Performance or First Steps Wellness Centre in Regina. Exercise sessions will be completed in groups of 1-4 participants under the

supervision of a clinical exercise physiologist. The total time commitment is ~51 hours over 18-20 weeks (60-minute exercise three times per week for 12 weeks = 36 hrs; three sets of two 2-hr assessments = ~12 hrs; three blood samples = ~3 hrs).

We will also request that you provide us with a hard copy of your clinical MRI results, obtained by you through your digital health records (i.e., MySaskHealthRecord) dating back to your initial MS diagnosis; however, you are not required to provide us with this information to be a participant in the study.

What are the benefits of participating in this study?

There is no direct benefit to you for participating in this study. Nevertheless, past research suggests that exercise similar to that which will be performed as part of the study is generally beneficial for management of many symptoms related to MS. Also, following completion of the study you will be provided with a free registration in one term of the Enrich Neurorehabilitation Program, a community exercise program for people with neurological conditions offered at the University of Regina Centre for Health, Wellness and Performance. Overall, it is hoped that findings made in this study will inform the development of specific exercise recommendations that target improvements in central nervous system function in people with MS.

What are the possible risks and discomforts?

The exercise sessions and assessments of fitness and movement abilities could plausibly result in fatigue, muscle soreness, and joint or skeletal injury. For example, during the fitness assessments, you will be asked to stand up repeatedly over 30 seconds to evaluate lower-limb strength and to exercise on a recumbent stepper until you feel like you need to stop. As fatigue is known to be a common challenge for people with MS, the study team will ask you regularly about your level of fatigue. Rest will be provided and sessions halted early or re-scheduled as needed and on your request. Qualified study staff certified in emergency first-aid and CPR will be present to monitor all sessions and will check in with you regularly to ensure that you are comfortable.

The following paragraphs outline the possible adverse effects experienced from the placement of sticky sensors on the skin (i.e., electrodes) and from the TMS technique mentioned above. Collectively, the potential discomfort experienced from the electrodes are more common but transient, while the side effects produced by TMS are rare. Nonetheless, all of the adverse effects described are generally uncommon.

You may get a skin rash under the sticky sensors used for measuring electrical activity in muscles. Rashes usually clear up within 2-3 hours of removing the sensors. Safety standards for the application of TMS have been developed and will be followed by trained operators during this study to minimize the risk. In accordance with these standards, the TMS machine will always be run at a rate and a frequency that are known to be safe. Related to TMS, there is a rare, but potential, risk of provoking a seizure in people with a history of seizures (e.g. epilepsy).

TMS can be delivered either one stimulus at a time (i.e., single-pulse TMS) or in repeated trains of stimuli (i.e., repetitive TMS). When using high frequency repetitive TMS, which will **NOT** be used in this study, there is a low risk of seizure induction (i.e., than 1%) in

individuals without epilepsy. In this study you will receive single-pulse TMS, which is generally not considered to provide a risk of seizure induction. Nevertheless, you will not be eligible to participate in this study if you have a history of seizures.

There is a risk of headache, scalp pain, toothache or scalp numbness associated with TMS. Each of these side effects is transient (i.e., does not last more than 2-3 hours). The clicks associated with TMS are somewhat loud and could potentially damage your hearing. To minimize this risk you will be asked to wear earplugs throughout the testing session however transient hearing loss remains possible.

When providing blood samples, you will feel the poke of the needle being inserted into your arm. You may experience light bruising in the region of the arm where the blood is drawn, but this typically resolves within 24 hours. There is also a more serious risk of infection associated with blood draws, however, these samples will be conducted by qualified personnel using sterile equipment.

It is possible that you may experience the listed discomforts or adverse effects after leaving the study site or facility (e.g., muscle soreness or headache). If this occurs you should seek immediate care as required and contact the Principal Investigator as soon as possible.

What happens if I decide to withdraw?

Your participation in this research is voluntary. You may withdraw from this study at any time by notifying any member of the research team through any means (e.g., face-to-face, phone, email). You do not have to provide a reason. Your relationships with the researchers or the university will not be affected. If you withdraw from the study before March 31, 2024, your data will be removed from all analyses and destroyed; otherwise it will be retained in the analysis.

What happens if something goes wrong?

Staff trained in emergency first-aid and CPR will be available at all times during the study sessions. In the rare case of a medical emergency related to the study but not occurring directly within a study session, you should seek immediate care and, as soon as possible, notify the Principal Investigator. Necessary medical treatment will be made available at no cost to you. By signing this document, you do not waive any of your legal rights against the investigators or anyone else.

What will the study cost me?

You will not be charged for any research-related procedures. You will not be paid for participating in this study. Arrangements will be made to cover parking expenses for all study sessions.

Will my participation be kept confidential?

In Saskatchewan, the Health Information Protection Act (HIPA) defines how the privacy of your personal health information must be maintained so that your privacy will be

respected. Your name and any potentially identifying information will not be attached to any information, nor mentioned in any study report, nor be made available to anyone except the research team. We will inform you directly if any findings obtained through our study procedures are considered to warrant medical concern. It is the intention of the research team to publish results of this research in scientific journals and to present the findings at related conferences and workshops, but your identity will not be revealed. Nevertheless, your participation in this study is not entirely anonymous. The research team, other participants, and persons engaging in activities at the venues of exercise program delivery will/may know that you are participating in a research study. Finally, the research team will employ measures to safeguard the confidentiality of group interactions during exercise program delivery, but cannot guarantee that other members of the exercise program group will do so. Please respect the confidentiality of the other members of the exercise program group by not disclosing the contents of the group interactions outside the group, and be aware that others may not respect your confidentiality.

Will I be informed of the research findings?

A summary of research findings will be available on the OURspace repository at the U of R library by (https://ourspace.uregina.ca) December 2024.

Who do I contact if I have questions about the study?

If you have questions concerning the study you can contact Dr. Cameron Mang at 306-585-4066.

If you have any questions about your rights as a research participant or concerns about this study, you may contact the Chair of the University of Regina Research Ethics Board at (306) 585-4775 or email research.ethics@uregina.ca. Out of town participants may call collect. The Research Ethics Board is a group of individuals (scientists, ethicists, and members of the community) that provide an independent review of human research studies. This study has been reviewed and approved on ethical grounds by the University of Regina Research Ethics Board as of April 14, 2022.

Consent statement

- I have read (or someone has read to me) the information in this consent form.
- I understand the purpose and procedures and the possible risks and benefits of the study.
- I was given sufficient time to think about it.
- I had the opportunity to ask questions and have received satisfactory answers.
- I am free to withdraw from this study at any time for any reason and the decision to stop taking part will not affect my future relationships at the university.
- I have been informed that this study will provide no direct benefits to me.
- I give permission for the use and disclosure of my de-identified personal health information collected for the research purposes described in this form.
- I understand that by signing this document I do not waive any of my legal rights.
- I will be given a signed and dated copy of this consent form.

❑ I agree to participate in this study:

❑ Check this box if you give consent and would like to be contacted about participating in future research projects in the Neuroplasticity and Neurorehabilitation Lab. If you do not check this box, then your contact information will be deleted after study completion.

Please tick a box below and provide your information to indicate whether you would prefer that the research team maintain contact with you via phone or email. Note: Email is not considered a secure form of communication. All email communication would have the phrase “MS Exercise CNS” in the subject line.

❑ Phone

or

❑ Email:

Printed name of participant: ____

Signature ________________________ Date______________________________

Printed name of person obtaining consent: ____________________________________

Signature ________________________ Date______________________________
